# Society Meetings

**Published:** 1898-09

**Authors:** 


### SOCIETY MEETINGS.

Officers of the Buffalo Academy of Medicine.—Session of 1898-1899.—President, Dr. Roswell Park; secretary, Dr. Thomas F. Dwyer; treasurer, Dr. Charles S. Jewett; trustees, Dr. B. G. Long (three years), Dr. Marcel Hartwig (two years), Dr. DeLancey Rochester (one year).

The vice-presidents by virtue of being chairmen of the various sections are: Dr. William G. Ring, chairman section on medicine; Dr. John Parmenter, chairman section on surgery; Dr. William G. Taylor, chairman section on obstetrics; Dr. A. L. Benedict, chairman section on pathology.

The council is therefore composed of the aforementioned officers, the president of the academy of the previous year, Dr. Lucien Howe, and the secretaries of the sections, who are: Dr. Fridolin Thoma, secretary section on medicine; Dr. J. Henry Dowd, secretary section on surgery; Dr. N. G. Russell, secretary section on obstetrics; Dr. A. G. Bennett, secretary section on pathology.

PROGRAM, 1898-1899.

September 6th.—Surgery.—Coöperation of physician and surgeon in abdominal cases, A. L. Benedict, M. D. Duties of army medical officers during active campaigning, William Warren Potter, M. D., Brevet Lieut.-Col. U. S. V.

September 13th.—Medicine.—Quarterly meeting of Academy.—Chronic joint affections, commonly called rheumatism, Charles G. Stockton, M. D. Rheumatism of childhood, B. G. Long, M. D.

September 20th.—Pathology.—Subject to be announced, M. V. Ball, M. D., Warren, Pa.

September 27th.—Obstetrics.—Some common mistakes in gynecology, M. D. Mann, M. D.

October 4th.—Surgery.—Concussion of the brain, Roswell Park, M. D. On the use of chloroform in adenoid operation, F. W. Hinkel, M. D.

October 11th.—Medicine.—Cardiac diseases: Subject to be announced, Allen A. Jones, M. D. Remarks on prognosis and cause of death in cardiac diseases, John H. Pryor, M. D. Serious heart disease without rheumatism, A. L. Benedict, M. D.

October 18th.—Pathology.—Devitalised teeth, W. C. Barrett, M. D.

October 25th.—Obstetrics.—Purulent ophthalmia, Lucien Howe, M. D.

November 1st.—Surgery.—Gonorrhea, W. H. Heath, M. D. Surgical kidney, M. Hartwig, M. D.

November 8th.—Medicine.—The pathogeny of an epileptic fit, L. Pierce Clark, M. D., Craig Colony, Sonyea, N. Y. Fränkel's movements in the treatment of locomotor ataxia, William C. Krauss, M. D.; discussion by Floyd S. Crego, M. D., and James W. Putnam, M. D.

November 15th.—Pathology.—Carcinoma of the duodenum, C. D. Aaron, M. D., Detroit, Michigan. Pathology of vertigo, Charles G. Stockton, M. D.

November 22d.—Obstetrics.—Multiple pregnancy, Thomas F. Dwyer, M. D.

December 6th.—Surgery.—Quarterly meeting of Academy.—Subject to be announced, N. Jacobson, M. D., Syracuse, N. Y.

December 13th.—Medicine.—Treatment of cases of pulmonary tuberculosis that cannot leave home, DeLancey Rochester, M. D. Essential asthenia, George N. Jack, M. D., Depew, N. Y.

December 20th.—Pathology.—The tests of renal insufficiency by methylene blue and iodine, Richard C. Cabot, M. D., Boston, Mass. Exhibition of lantern slides of various skin affections, Grover W. Wende, M. D.

December 27th.—Obstetrics.—Subject to be announced, C. C. Frederick, M. D.

January 3d.—Surgery.—Burns, scalds and frost bites, A. E. Diehl, M. D. Colles fracture, Edward M. Dooley, M. D.

January 10th. Medicine.—Insanity. Arthur W. Hurd, M. D., Harry A. Wood, M. D., Henry P. Frost, M. D., Helene Kuhlmann, M. D., C. J. Patterson, M. D.

January 17th.—Pathology.—The pathology of catarrhal deafness, F. W. Hinkel, M. D.; discussion opened by H. Y. Grant, M. D.

January 24th.—Obstetrics.—Subject to be announced, M. A. Crockett, M. D.

February 7th.—Surgery.—Incarcerated inguinal hernia, treatment by nonoperative methods, W. C. Phelps, M. D. Treatment of stricture of nasal duct by electricity, B. H. Grove, M. D.

February 14th.—Medicine.—The status of pharmacy today and its possible improvements, Eli H. Long, M. D.; discussion by Allen A. Jones, M. D., Willis G. Gregory, M. D., Mr. George Reimann, Mr. J. A. Lockie, H. C. Buswell, M. D., and G. W. York, M. D.

February 21st.—Pathology.—Pathology of alcoholism, J. W. Grosvenor, M. D.; discussion opened by H. R. Hopkins, M. D. Intraocular tumors, B. H. Grove, M. D.

February 28th.—Obstetrics.—The epoch of maternity and its influence on the mind, William P. Spratling, M. D., Craig Colony, Sonyea, N. Y.

March 7th.—Surgery.—Foreign bodies as ligatures, C. P. Smith, M. D. Subject to be announced, John W. Whitbeck, M. D., Rochester, N. Y.

March 14th.—Medicine.—Differentiation of renal diseases, Thomas B. Carpenter, M. D. Acute Bright's disease, J. Henry Dowd, M. D. Chronic Bright's disease, Albert H. Woehnert, M. D. Primary nephritis (infantile), G. A. Himmelsbach, M. D.

March 21st.—Quarterly meeting of Academy.—Subject to be announced, Frederick Peterson, M. D., New York.

March 28th.—Obstetrics.—Subject to be announced, H. E. Hayd, M. D.

April 4th.—Surgery.—Acute osteo-myelitis, Roswell Park, M. D.

April 11th.—Medicine.—Diseases of the skin, Alfred E. Diehl, M. D., Grover W. Wende, M. D., Frank J. Thornbury, M. D.

April 18th.—Pathology.—Our present knowledge of the inflammatory processes, H. R. Gaylord, M. D. Subject to be announced, Prof. Charles W. Dodge, Rochester, N. Y.

April 25th.—Obstetrics.—Subject to be announced, Charles E. Congdon, M. D.

May 2d.—Surgery.—Subject to be announced.

May 9th.—Medicine.—The use of alcohol in the treatment of diseases, Joseph W. Grosvenor, M. D., Henry R. Hopkins, M. D., A. L. Benedict, M. D., E. L. Frost, M. D., Sydney A. Dunham, M. D.

May 16th.—Pathology.—Pathology and diagnosis of certain spinal diseases, Floyd S. Crego, M. D. Pathology of epilepsy, W. C. Krauss, M. D.

May 23d.—Obstetrics.—Subject to be announced, Stephen Y. Howell, M. D.

June 6th.—Surgery.—Cystitis and urinary infection, J. Henry Dowd, M. D.

June 13th.—Annual meeting of Academy.

June 20th.—Pathology.—Subject to be announced, Herbert M. Hill, Ph. D.

June 27th.—Obstetrics.—Quarterly meeting of Academy.—The umbilical cord, P. W. Van Peyma, M. D. Proper management of an obstetrical case, H. C. Buswell, M. D.

---

The New York State Medical Association will hold its next annual meeting in the City of New York, October 18, 19 and 20, 1898. An interesting preliminary program has already been published and the meeting will undoubtedly enjoy its usual success.

---

The Medical Association of Central New York will hold its thirty-first annual meeting at Auburn, N. Y., Tuesday, October 18, 1898, under the presidency of Dr. William C. Krauss, of Buffalo. The secretary, Dr. Charles A. Van der Beek, of Rochester, is already in the field with requests for papers. This will be the first meeting of the association in Auburn and the local committee bespeaks a full attendance and promises an occasion of much interest.

---

The American Public Health Association will hold its twenty-sixth annual meeting at Ottawa, Canada, September 27-30, 1898, under the presidency of Prof. Charles A. Lindsley, M. D., of New Haven, Conn. Dr. C. O. Probst, Columbus, O., is the secretary, to whom all communications relating to papers, etc., should be addressed.

The American Electro-therapeutic Association will hold its next annual meeting at Buffalo, September 13, 14 and 15, 1898, under the presidency of Dr. Charles R. Dickson, of Toronto. Dr. John Gerin, of Albany, is the secretary, to whom communications should be addressed concerning the meeting.

The Southern Surgical and Gynecological Association will hold its next annual meeting at Memphis, Tenn., Tuesday, Wednesday and Thursday, November 8, 9 and 10, 1898, under the presidency of Dr. Richard Douglas, of Nashville. The secretary, Dr. Wm. E. B. Davis, of Birmingham, Ala., has begun the campaign and the auspices are favorable for an excellent meeting.

